# Retinal function determined by flicker ERGs before and soon after intravitreal injection of anti-VEGF agents

**DOI:** 10.1186/s12886-019-1129-7

**Published:** 2019-06-17

**Authors:** Gaku Terauchi, Kei Shinoda, Hiroyuki Sakai, Makoto Kawashima, Celso Soiti Matsumoto, Atsushi Mizota, Yozo Miyake

**Affiliations:** 1 Department of Ophthalmology, Teikyo University School of Medicine, 2-11-1 Kaga, Itabashi-ku, Tokyo, 173-8605 Japan; 2 Department of Ophthalmology, Saitama Medical University Faculty of Medicine, 38 Moro-hongo, Moroyama, Iruma-gun, Saitama, 350-0495 Japan; 3 Matsumoto Eye Clinic, 50-2 Takagaki, Awa-cho, Awa-shi, Tokushima, 771-1705 Japan; 4 Department of Ophthalmology, Aichi Medical University School of Medicine, 1-1 Yazakokarimata, Nagakute, Aichi 480-1195 Japan

**Keywords:** Aflibercept, Age-related macular degeneration, Electroretinogram, Intravitreal injection, Macular edema, Ranibizumab, Retinal vein occlusion, Vascular endothelial growth factor

## Abstract

**Background:**

To evaluate the retinal function before and soon after an intravitreal injection of an anti-vascular endothelial growth factor (anti-VEGF) agents.

**Methods:**

Seventy-nine eyes of 79 patients that were treated by an intravitreal injection of an anti-VEGF agent for age-related macular degeneration (AMD), diabetic macular edema (DME), or retinal vein occlusion (RVO) with macular edema (ME) were studied. The RETeval® system was used to record 28 Hz flicker electroretinograms (ERGs) from the injected and non-injected eyes before (Phase 1, P1), within 2 h after the injection (P2), and 2 to 24 h after the injection (P3). Patients were grouped by disease or by the injected agent and compared. The significance of the changes in the implicit times and amplitudes was determined by *t* tests.

**Results:**

The amplitudes were not significantly different at the three phases. The implicit time of the injected eye was 31.2 ± 3.2 msec at P1, and it was not significantly different at P2 (31.7 ± 3.1 msec) but it was significantly longer at P3 (32.2 ± 3.3 msec, *P* < 0.01, ANOVA for both). The implicit time in the non-injected fellow eye was 30.5 ± 3.3 msec at P1, and it was significantly longer at P2 (31.1 ± 3.2 msec) and phase 3 (31.3 ± 3.4 msec, *P* < 0.01, ANOVA for both).

**Conclusions:**

The results indicate that an intravitreal anti-VEGF injection will increase the implicit times not only in the injected eye but also in the non-injected eye soon after the intravitreal injection.

## Background

Intravitreal injections are used to deliver drugs and gases to the retina and choroid to treat various eye diseases. Since pegaptanib, an anti-vascular endothelial growth factor (anti-VEGF) aptamer, was approved by the United States Food and Drug Administration in 2004, the number of intravitreal injections has been increasing. Ranibizumab, aflibercept, and bevacizumab are commonly used as anti-VEGF agents [1–10], and they were originally used to treat eyes with age-related macular degeneration (AMD) [1–4]. Their use has expanded and they are being used to treat diabetic macular edema (DME) [5–10], retinal vein occlusion (RVO) with macular edema (ME) [11–14], and other vascular-related retinal diseases. Many clinical trials have reported on their effectiveness of these agents [1–14]. In addition, the number of intravitreal injections of ocriplasmin [15] and steroids has increased, and intravitreal injections have become a relatively common procedure.

There are, however, serious side effects such as endophthalmitis, vascular occlusions, retinal tears, and rhegmatogenous retinal detachment that can develop after an intravitreal injection [16–19]. There are also systemic complications such as cerebral and myocardial infarctions that can develop after intravitreal injections of these agents [20]. These findings indicate that these agents can have systemic effects and attention needs to be paid to these possibilities.

Retinal functions can be evaluated by electroretinography (ERG) and can be performed not only by the conventional ERG systems [21–23] but also by a new RETeval® system. This RETeval® system consists of a handheld, portable ERG device that includes stimulating and recording elements [21, 24, 25]. The ERGs are picked-up by a skin electrode array that is fixed to the lower eyelid. Skaat et al., evaluated the retinal function by conventional ERGs after intravitreal injection of bevacizumab into eyes with AMD and the non-injected normal fellow eye. The ERGs were recorded before and one month after the intravitreal injection [26]. They reported that the a-wave amplitudes of both the scotopic and photopic ERG in injected eyes were significantly larger than that of the controls. To focus on the retinal function immediately after injection of an anti-VEGF agent, we recorded ERGs before and within 2 h and within 24 h of the intravitreal injection. Such recordings have not been reported because the use of a contact lens for the recording electrodes would have raised the risk of infections and injury to the eye. The RETeval® system uses skin electrodes placed on the lower eyelid, and can be used to assess the function of both eyes soon after any intraocular procedures.

Thus, the purpose of this study was to evaluate the retinal function before and at < 2 h and between 2 and 24 h after an intravitreal injection of different anti-VEGF agents. We determined the changes in the retinal function of the injected eyes and the non-injected fellow eye.

## Patients and Methods

### Patients

Seventy-nine eyes of 79 patients who had received an intravitreal injection of an anti-VEGF agent at the Teikyo University Hospital in Tokyo, Japan from June 2014 to August 2015 were studied. They had received the anti-VEGF agent for AMD (*n* = 37), DME (*n* = 24), or RVO with ME (*n* = 18). The injected anti-VEGF agent was ranibizumab or aflibercept (Table 1). If the patients had been injected more than once within the study period, only the initial data were used. In patients who had been injected in both eyes during the study period, only the data collected after the first injection was included. The exclusion criteria included eyes with severe retinal diseases in which the ERGs were non-recordable from either eyes, cases where the injected eye had had laser treatment, and cases where the fellow eye underwent intravitreal anti-VEGF injection within one month of the beginning of this study.

**Table 1:** Number of eyes listed by disease and by injected agent

	AMD	DME	RVO with ME	total
ranibizumab	18	18	14	50
aflibercept	19	6	3	28
total	37	24	17	78

Patient breakdown by disease and by injected agent

*AMD* age-related macular degeneration;

*DME* diabetic macular edema;

*RVO with ME* retinal vein occlusion with macular edema

### Ophthalmic examinations

Flicker ERGs were recorded three times from both eyes with the RETeval® system (Mayo, Inazawa, Japan); the first recording was made before the injection of an anti-VEGF agent (Phase 1), the second was within 2 h after the injection (Phase 2), and the third was 2 to 24 h after the injection (Phase 3). The components of the RETeval® system have been described in detail [21, 24, 25, 27]. Recordings were made under room light conditions after mydriasis, and the flicker ERGs were picked-up by a sensor strip skin electrode array (Sensor Strip; LKC Technologies, Inc.) that was affixed 2 mm at the margin of the lower eyelid of both eyes. The strips included the active, reference, and ground electrodes. The electrical potentials were direct coupled (DC) -amplified and digitized with a 2 kHz sampling rate. The data resolution was 24 bits for ±0.6 V which is equal to approximately 0.07 μV.

**Fig. 1:**
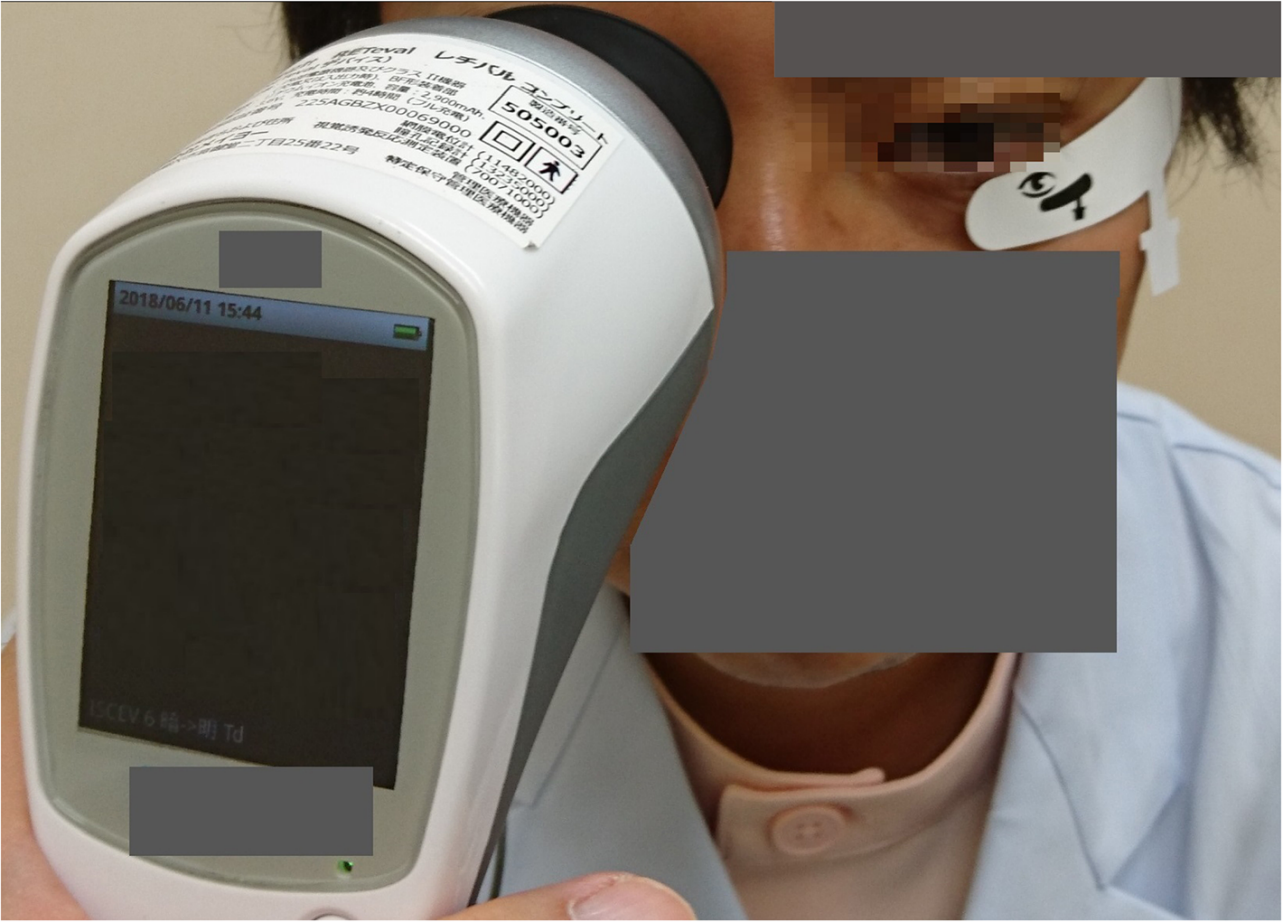
Image of measuring 28 Hz flicker ERG using RETeval®. The image showed that demonstration of the measuring 28 Hz flicker ERG used by RETeval® under the room lighting

A mini Ganzfeld dome was placed in front of the eye, and stimulated with white stimuli (CIE 1931 chromaticity, x.0.33, y.0.33). The white stimuli were created by a combination of three colored light emitting diodes (LEDs; red 622 nm; green, 530 nm; blue, 470 nm; CLV6A-FKB; Cree, Inc., Durham, NC, USA). The luminance of the stimuli were 3 cd·s/m^2^ flash with a 30 cd/m^2^ background light (ISCEV standard). The frequency of the flicker stimulus was 28.306 Hz (period of 35.328 ms), and the pulse duration was less than 1 msec (confirmed by recording the LED responses with a photodiode). The flicker ERG recording time ranged from 5 to 15 s depending on the reliability of the results which was assessed by estimating the standard error of the mean of the implicit time from all the sweeps. Thus, the ERGs that were elicited by 141 to 425 flashed were analyzed for each recording. If the subject blinked, as determined by the infrared camera, the data were not used in the analysis by the RETeval® system. The amplitudes and implicit times of the fundamental component were automatically measured and displayed by the RETeval® system using special algorithm with discrete fourier transformation (DFT) and cross-correlation analysis [28]. Because the response to a periodic stimulus is composed of sinusoidal components that are multiples of the stimulus frequency, it is possible to reconstruct a less noisy version of the raw flicker ERG waveform by determining the amplitude and phase of each of the harmonics and summing them [28]. The patients were instructed to look at a fixation point within the dome, and the fixation was monitored by an infrared camera (Fig. 1). The implicit times and amplitudes of the flicker ERGs were automatically analyzed by the software integrated in the RETeval® system.

Visual acuity measurement, slit lamp and fundoscopic examinations, and OCT scanning were performed before injection. OCT scanning was done in 78 eyes before the ERG recording, and in 57 eyes at Phase 2, and 57 eyes at Phase 3. The decimal visual acuities were converted to the logarithm of the minimum angle of resolution (logMAR) units for the statistical analysis. The mean foveal thickness at 1 mm diameter of the ETDRS 9 sector was measured by OCT.

### Intravitreal injections

The intravitreal injections were done under topical 4% lidocaine anesthesia. The conjunctival sac was disinfected by 10% povidone-iodine and 0.05% chlorhexidine gluconate, and a sterile lid speculum was used. After the ocular surface was prepared, aqueous humor was aspirated from the limbus with a 30-gauge needle. The ocular surface was disinfected again with 0.25% povidone-iodine solution, and then 0.05 ml of the anti-VEGF agent was injected into the vitreous through the pars plana with a 30-gauge needle. Antibiotics eye drops and ointment were applied and a sterile eyepatch was placed over the eye.

The patients were grouped and compared by the type of disease or by the type of anti-VEGF agent injected. For statistics, repeated measures tests and paired *t* tests were used to determine whether the changes in the implicit times and amplitudes among the groups were significant. A *P* value of *P* < 0.05 was taken to be significant.

## Results

We excluded cases that did not have a complete set of recordings during the three phases, and also those whose amplitudes were too small to be analyzed by the RETeval® system. In the end, 79 cases were analyzed and 9 cases were excluded. There were no cases in which both eyes were injected at the same examination time.

The mean age of the patients was 68.9 ± 12.6 years, and they were made up of 44 men and 35 women (Tables 1 and 2). The mean ± standard deviation of the BCVA before the injection was 0.59 ± 0.48 logMAR units in the injected eye and 0.23 ± 0.53 logMAR units in the non-injected fellow eye. The difference in the BCVA was significant (*P* = 0.22 × 10^− 4^). The mean foveal thickness in the injected eye was 418.6 ± 151.8 μm at Phase 1, 325.5 ± 186.8 μm at Phase 2, and 387.6 ± 344.7 μm at Phase 3. The thickness in the non-injected fellow eye was 292.6 ± 123.1 μm at Phase 1, 299.0 ± 109.9 μm at Phase 2, and 304.6 ± 92.8 μm at Phase 3. The demographics of the injected eyes according to the retinal disease and injected agents are shown in Table 1.

**Table 2:** Results of all cases

*n* = 79 (male: 44, female: 35)				
mean age		68.9 ± 12.6 y.o.		
injected eye	Phase 1	Phase 2	Phase 3	repeated measure test
implicit time	31.2 ± 3.2	31.7 ± 3.1^a^	32.2 ± 3.3^a^	*p* < 0.00001
(msec.)		*p* = 0.00034	*p* < 0.00001	
amplitude	8.7 ± 5.7	7.9 ± 6.1	9.5 ± 8.9	*p* = 0.12
(uV)		*p* = 0.5	*p* = 0.92	
non-injected eye	Phase 1	Phase 2	Phase 3	repeated measure test
implicit time	30.5 ± 3.3	31.1 ± 3.18^a^	31.3 ± 3.4^a^	*p* = 0.000018
(msec.)		*p* = 0.0084	*p* = 0.00002	
amplitude	9.4 ± 6.5	9.2 ± 6.7	9.5 ± 6.2	*p* = 0.72
(uV)		*p* = 1	*p* = 1	

The results of 28 Hz flicker ERG components including all patients before classification by disease of by injected agent. The ERGs were recorded before the injection (Phase 1), within 2 h after the injection (Phase 2), and 2 to 24 h after the injection (Phase 3)

mean ± standard deviation

^a^; significant difference by the repeated measure test and by the post hoc test compared to Phase 1

### Electroretinographic findings

**Fig. 2:**
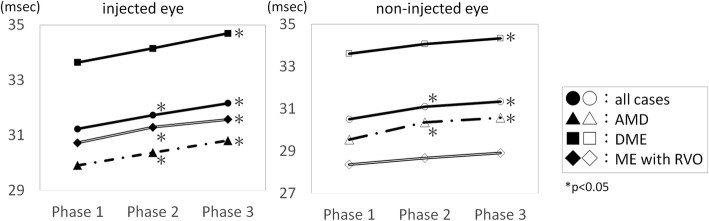
Implicit times of each eye segregated by disease. The implicit time of each eye segregated by disease before (Phase1), within 2 h after the injection (Phase 2), and 2 to 24 h after the injection (Phase 3). The implicit times were significantly longer at Phase 2 and Phase 3 than at Phase 1. *; *P* < 0.05. AMD, age-related macular degeneration. DME, diabetic macular edema. RVO with ME, retinal vein occlusion with macula edema

**Fig. 3:**
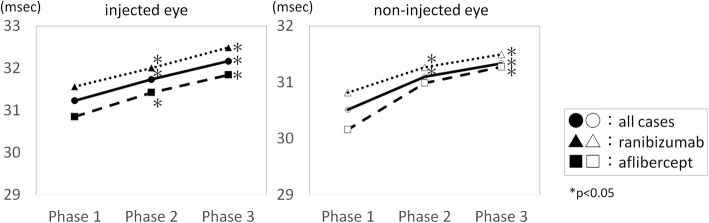
Implicit time of each eye segregated by the injected agent. The implicit time of each eye segregated by injected agent before (Phase 1), within 2 h after the injection (Phase 2), and 2 to 24 h after the injection (Phase 3). The implicit times were longer at Phase 2 and Phase 3 than at Phase 1 as the baseline. *; *P* < 0.05

All cases (Figs. 2 and 3; Table 2).

The repeated measures tests of the implicit times showed significant changes in the injected eye (*P* < 0.1 × 10^− 5^) and the non-injected fellow eye (*P* = 0.18 × 10^− 5^). The differences in the implicit times between Phase 1 and Phase 2, and between Phase 1 and Phase 3 were significant in both the injected and non-injected fellow eyes.

The differences in the amplitudes were not significant in the repeated measures tests and the paired *t* tests at any Phase.

### Subgroup analysis by disease (Fig. 2; Table 3)

In the AMD group, the repeated measures tests of the implicit time showed significant changes in the injected eye (*P* = 0.85 × 10^− 4^) and the non-injected fellow eye (*P* = 0.97 × 10^− 3^). The difference in the implicit times between Phase 1 and Phase 2, and between Phase 1 and Phase 3 were significant in both the injected and non-injected eyes.

**Table 3:** Results of analyses by disease

3–1. ERG responses before and after injection in eyes with AMD				
AMD *n* = 37 (male, 24; female, 13)				
mean age	74.1 ± 8.6 y.o.			
injected eye	Phase 1	Phase 2	Phase 3	repeated measure test
implicit time	29.9 ± 1.8	30.4 ± 1.6^a^	30.8 ± 2.14^a^	*p* = 0.00009
(msec.)		*p* = 0.025	*p* = 0.00001	
amplitude	9.0 ± 5.7	8.1 ± 6.4	9.0 ± 6.3	*p* = 0.484
(uV)		*p* = 1	*p* = 1	
non-injected eye	Phase 1	Phase 2	Phase 3	repeated measure test
implicit time	29.5 ± 2.5	30.4 ± 2.1^a^	30.6 ± 2.4^a^	*p* = 0.00097
(msec.)		*p* = 0.023	*p* = 0.0017	
amplitude	10.1 ± 7.2	9.6 ± 7.3	9.8 ± 6.2	*p* = 0.853
(uV)		*p* =1	*p* =1	
3–2. ERG responses before and after injection in eyes with DME				
DME *n* = 24 (male, 14; female, 10)				
mean age	59.9 ± 11.3 y.o.			
injected eye	Phase 1	Phase 2	Phase 3	repeated measure test
implicit time	33.6 ± 3.2	34.2 ± 3.2	34.7 ± 3.1^a^	*p* = 0.00036
(msec.)		*p* = 0.19	*p* = 0.0003	
Amplitude	8.0 ± 5.9	7.1 ± 5.8	7.6 ± 5.9	*p* = 0.45
(uV)		*p* = 0.94	*p* = 0.96	
non-injected eye	Phase 1	Phase 2	Phase 3	repeated measure test
implicit time	33.6 ± 3.1	34.1 ± 3.1	34.3 ± 3.4^a^	*p* = 0.0493
(msec.)		*p* = 0.327	*p* = 0.014	
Amplitude	7.5 ± 5.1	7.2 ± 5.5	7.8 ± 5.6	*p* = 0.495
(uV)		*p* = 1	*p* = 1	
3–3. ERG responses before and after injection in eyes with ME associated with RVO				
RVO with ME *n* = 18 (male, 6; female, 12)				
mean age	70.1 ± 14.9 y.o.			
injected eye	Phase 1	Phase 2	Phase 3	repeated measure test
implicit time	30.7 ± 3.7	31.3 ± 3.4	31.6 ± 3.79^a^	*p* = 0.00692
(msec.)		*p* = 0.1125	*p* = 0.0027	
Amplitude	8.9 ± 5.7	8.5 ± 5.8	12.9 ± 14.7	*p* = 0.214
(uV)		*p* = 1	*p* = 0.68	
non-injected eye	Phase 1	Phase 2	Phase 3	repeated measure test
implicit time	28.4 ± 1.9	28.6 ± 2.0	28.9 ± 2.3	*p* = 0.231
(msec.)		*p* = 0.85	*p* = 0.53	
amplitude	10.5 ± 6.4	10.8 ± 6.5	11.2 ± 6.7	*p* = 0.573
(uV)		*p* = 1	*p* = 0.96	

The results of 28 Hz flicker electroretinogram components classified by disease. The ERGs were recorded before the injection (Phase 1), within 2 h after the injection (Phase 2), and 2 to 24 h after the injection (Phase 3)

mean ± standard deviation

^a^; significant difference by the repeated measure test and by the post hoc test compared with Phase 1

*AMD* age-related macular degeneration;

*DME* diabetic macular edema;

*RVO with ME* retinal vein occlusion with macular edema

The differences in the amplitudes were not significant in the repeated measures test or the paired *t* test at any Phase for the eyes with AMD.

In the DME group, the repeated measures test of the implicit times showed significant changes in the injected eye (*P* = 0.36 × 10^− 3^) and the non-injected fellow eye (*P* = 0.049). The difference in the implicit times between Phase 1 and Phase 3 was significant in the injected and non-injected fellow eyes. No significant difference was observed between Phase 1 and Phase 2 in the injected and non-injected fellow eyes.

The differences in the amplitudes were not significant in the repeated measures tests or the paired *t* test at any Phase.

In the RVO with ME group, the repeated measures test of the implicit times showed significant changes in the injected eye (*P* = 0.69 × 10^− 2^). Significant differences between Phase 1 and Phase 2, and between Phase 1 and Phase 3 were observed only in the injected eyes.

The differences in the amplitudes were not significant in the repeated measures test or the paired *t* test at any Phase.

### Subgroup analysis for different injected agents (Fig. 3, Table 4)

Fifty eyes of 50 patients were injected with ranibizumab, 28 eyes of 28 patients were injected with aflibercept, and one eye of one patient was injected with bevacizumab (Table 1). First, we compared the results of the eyes injected with ranibizumab to that of eyes injected with aflibercept.

**Table 4:** Results of analyses by injected agent

4–1. ERG responses before and after injection of ranibzumab.				
ranibizumab *n* = 50 (male, 22; female, 28)				
mean age		68.1 ± 13.5 y.o.		
injected eye	Phase 1	Phase 2	Phase 3	repeated measure test
implicit time	31.6 ± 3.45	32.0 ± 3.45^a^	32.5 ± 3.57^a^	*p* < 0.000001
(msec.)		*p* = 0.012	*p* < 0.000001	
amplitude	7.18 ± 5.34	6.68 ± 5.60	8.21 ± 9.97	*p* = 0.377
(uV)		*p* = 1	p = 1	
non-injected eye	Phase 1	Phase 2	Phase 3	repeated measure test
implicit time	30.8 ± 3.24	31.3 ± 3.51	31.5 ± 3.62^a^	*p* = 0.0029
(msec.)		*p* = 0.062	*p* = 0.0052	
amplitude	8.21 ± 6.00	7.32 ± 4.79	8.31 ± 5.54	*p* = 0.144
(uV)		*p* = 0.45	p = 1	
4–2. ERG responses before and after injection of aflibercept.				
aflibercept *n* = 28 (male: 21, female: 7)				
mean age		70.1 ± 11.0 y.o.		
injected eye	Phase 1	Phase 2	Phase 3	repeated measure test
implicit time	30.9 ± 2.43	31.4 ± 2.09^a^	31.8 ± 2.55^a^	*p* = 0.0032
(msec.)		*p* = 0.020	*p* = 0.000002	
amplitude	11.5 ± 5.41	10.0 ± 6.49	11.8 ± 6.45	*p* = 0.088
(uV)		*p* = 0.4	*p* = 1	
non-injected eye	Phase 1	Phase 2	Phase 3	repeated measure test
implicit time	30.2 ± 3.33	31.0 ± 2.36	31.3 ± 2.85^a^	*p* = 0.0040
(msec.)		*p* = 0.17	*p* = 0.0039	
amplitude	11.7 ± 6.86	12.5 ± 8.23	11.8 ± 6.80	*p* = 0.511
(uV)		*p* = 1	*p* = 1	

The results of 28 Hz flicker electroretinogram components classified by injected agent. The ERGs were recorded before the injection (Phase 1), within 2 h after the injection (Phase 2), and 2 to 24 h after the injection (Phase 3)

mean ± standard deviation

^a^; significant difference by the repeated measure test and by the post hoc test compared with Phase 1

In the ranibizumab-injected group, the repeated measures test of the implicit time showed significant changes in the injected eye (*P* < 0.1 × 10^− 5^) and the non-injected fellow eye (*P* = 0.29 × 10^− 2^). Significant differences between Phase 1 and Phase 2, and between Phase1 and Phase 3 were observed in both the injected and non-injected eyes.

The differences in the amplitudes were not significant in the repeated measures tests or the paired *t* tests at any Phase.

In the aflibercept-injected group, the repeated measures tests of the implicit times showed significant changes in the injected eye (*P* = 0.32 × 10^− 3^) and the non-injected fellow eye (*P* = 0.39 × 10^− 2^). Significant differences between Phase 1 and Phase 2 and between Phase1 and Phase 3 were observed in the injected eyes, and significant difference was observed only between Phase1 and Phase 3 in the non-injected eyes.

The differences in the amplitudes were not significant in the repeated measures tests or the paired *t* tests at any Phase. None of the eyes had a serious complication during the course of this study.

## Discussion

The amplitudes of the flicker ERGs were not significantly different at the different phases in both eyes and all the subgroups classified by the injected agent or the disease. This is consistent with previous studies [21–23]. However, this lack of significant differences in the amplitudes of the flicker ERGs may be due to the relatively large variations in the amplitudes of the flicker ERGs.

On the other hand, the implicit times were significantly longer at Phase 2 and Phase 3 than at the baseline for each disease and for each injected agent except for Phase 2 in the DME group. This differs from the results of Yasuda et al. who reported that the implicit times of the flicker ERGs of the injected eyes were significantly shortened from 32.2 ± 2.6 msec to 30.6 ± 2.2 msec at one month after an intravitreal injection of ranibizumab in eyes with a central RVO [21]. They also reported that the implicit times of the injected eyes were significantly longer than that of the non-injected fellow eyes before the injection of ranibizumab. However, they did not compare the implicit times of the non-injected fellow eyes before and after the injection of ranibizumab in the affected eye. Holm et al. reported that the implicit time of the 30 Hz flicker full-field ERGs were significantly shorter at 4 weeks after the third monthly injection anti-VEGF agents in eyes with DME [22]. Gabriel et la reported that the implicit times and the amplitudes of all components of the full-field ERGs were not significantly different at 12 and 24 weeks after the intravitreal injection of ziv-aflibercept in eyes with diabetic retinopathy [23]. The major difference between these studies and our study was the time when the parameters of the ERGs were assessed, viz., our measurements were made within 24 h of the injection while the other studies made the measurements 4 to 12 weeks after the injection. It is known that it requires some time for any agents to exert their therapeutic effects such as on the visual acuity or the improvement of macular edema. In our study, we may have been able to detect the influence of the anti-VEGF agents on the retina at a very earlier phase rather than after the therapeutic effects of the agents had occurred. Januschowski et al. reported that no significant reduction in the amplitudes of the a- and b-waves of the isolated bovine retina perfused with aflibercept was observed at the end of the washout, but there were significant reductions in those directly after an exposure to aflibercept [29]. Myers et al. reported that the mean amplitude of the b-wave was significantly reduced 8 weeks after the intravitreal injection of the different anti-VEGF agents in normal rabbits [30]. Although their research was on rabbits, the ERGs were recorded at different post-injection times, and they showed a reduction in the b-wave amplitude. Our results are consistent with their conclusions that the anti-VEGF agent affected the retinal function adversely.

Our result showed that the differences in the implicit times of the non-injected fellow eye with aflibercept at Phase 2 was not significant in contrast to that with ranibizumab at Phase 2. This may be related to the differences in the time required for the agent to reach the fellow eye. Avery et al. reported that the serum concentration of ranibizumab peaked at 3 h after the intravitreal injection and aflibercept peaked at 1 day after the intravitreal injection [31, 32]. This difference in the peak times support our results of a time difference between the two agents.

In the perioperative period, we must consider the influence of the intraocular conditions that were changed by the intravitreal injection. There is a possibility that the postoperative intraocular pressure (IOP) may have had some influence on the ERG components. Miyake et al. reported a prolongation of the implicit time and a reduction in the amplitude on the intraoperative 30 Hz flicker ERGs during vitreous surgery [33, 34]. Yagura et al. reported a significant reduction in the amplitude and a prolongation of the implicit times of different components of the photopic ERGs after an intravitreal injection when a paracentesis was not performed [35]. They also reported that no significant differences were observed in the amplitudes and implicit times of almost all components of the photopic ERGs after an intravitreal injection followed by the aspiration of the aqueous humor by paracentesis. Although we did not measure the IOP, we believe that the influence of the IOP was minimal because the method of injecting the agents and IOP control were performed in the same way as Yagura et al. We measured the influence of the intravitreal injection of anti-VEGF agents on the fellow eyes. Although it is possible to investigate the agent concentration and VEGF activity in the injected eye, it is not ethically possible to collect specimens from the fellow eyes. Therefore, the concentration and VEGF activity in the fellow eye were not available. In contrast, our method has an advantage of being able to evaluate the effects of the agents non-invasively. We found that there was a significant delay or a trend in the delay of the implicit times in the fellow eyes just as in the injected eye. These results suggest that anti-VEGF agents enter the systemic circulation, and reach and affect the fellow eyes. Avery et al. reported that the serum concentration of anti-VEGF agents increased and the plasma concentration of free VEGF decreased after an intravitreal injection of anti-VEGF agents [31, 32]. These findings are consistent with the results of our study in term of the transmission into the systemic circulation.

Although anti-VEGF agents have become a standard treatment for AMD, DME, RVO with ME, and other retinal diseases, only limited information is available concerning the influence of the anti-VEGF agents on the fellow eye especially during the early periods. Further investigations on the long-term influence on the non-injected fellow eye are needed.

Our study has several limitations. One is that some of the patients had the disease bilaterally, and the non-injected fellow eye served as a control. We compared the changes relative to the baseline in the fellow eye as well their influence of the disease of the non-injected eye. However, the changes in retinal function on the non-injected fellow eye should be carefully interpreted. Further investigations on eyes with unilateral disease is necessary to strictly clarify the transmission of the effects of intravitreally injected agents on the healthy fellow eye.

Second, there may be other factors that might have affected the ERGs. For example, Horiguchi et al. reported that the vitreous temperature can affect the ERGs during vitrectomy [36]. However, we believe that the change in the vitreous temperature was probably minimal. Third, the sample size was relative small. When the eyes were classified into the different groups by agents or by diseases, the number decreased to less than 10 cases in some groups. It is necessary to collect more cases to assess the influence of each agent on the same disease or the influence on each disease with the same agent. Fourth, only the flicker ERG was recorded and no ERG under scotopic condition was recorded. This was because RETeval system enabled only flicker ERG, and because the repeated scotopic ERG recordings which needs at least 20 min of dark-adaptation for each eye would increase the patients’ discomfort. RETeval could record ERGs only one eye at time, and so recordings of both eyes need dark-adaptation twice. Because recent versions of the RETeval allow for scotopic and photopic ERG recordings, further study evaluating scotopic and photopic ERGs would be more informative. Taking these limitations into consideration, we still believe that this study is clinically important in that we could show the influence of the agent on the injected eye as well as non-injected fellow eye in the very early postoperative period.

## Conclusions

The results show that the implicit times of the flicker ERGs recorded with the RETeval system is prolonged not only in the injected eye but also in the non-injected fellow eye shortly after an intravitreal anti-VEGF agent injection. It is necessary to evaluate the long-term influence of anti-VEGF agents of the fellow eye.

## Data Availability

The datasets used andr analyzed during the current study are included. (full raw data list 20190511.excel).

## Abbreviations

AMD: Age-related macular degeneration
anti-VEGF: Anti-vascular endothelial growth factor
DME: Diabetic macular edema
ERG: Electroretinography
IOP: Intraocular pressure
ME: Macular edema
RVO: Retinal vein occlusion
U.S. FDA: the United States Food and Drug Administration
